# Supplementary material for: Bioproducts from Passiflora cincinnata Seeds: The Brazilian Caatinga Passion Fruit
Source: Foods. 2023 Jun 29;12(13):2525. doi: 10.3390/foods12132525 (PMC10340417; doi:10.3390/foods12132525)
Supplement: Supplementary file 1 [file foods-12-02525-s001.zip › foods-2438129-supplementary.pdf]

Bioproducts from *Passiflora cincinnata* seeds: The Brazilian Caatinga passion fruit

Carolina Cruzeiro Reis[1], Suely Pereira Freitas[1], Carolline Margot Albanez Lorentino[2], Thayssa da Silva Ferreira Fagundes[3,4], Virgínia Martins da Matta[5], André Luis Souza dos Santos[2], Davyson de Lima Moreira[3,6,*], Claudete Norie Kunigami[7], Eliane Przytyk Jung[7] and Leilson de Oliveira Ribeiro[7,*]

1 Laboratory of Vegetable Oil, Federal University of Rio de Janeiro, Rio de Janeiro 21941-909, Brazil; carolinacruzeiror@gmail.com (C.C.R.); freitasp@eq.ufrj.br (S.P.F.)

² Laboratory for Advanced Studies of Emerging and Resistant Microorganisms, Federal University of Rio de Janeiro, Rio de Janeiro 21941-902, Brazil; carolline.margot@gmail.com (C.M.A.L.); andre@micro.ufrj.br (A.L.S.d.S.)

³ Laboratory of Natural Products, Rio de Janeiro Botanical Garden Research Institute, Rio de Janeiro 22460-030, Brazil; thayssafagundes@id.uff.br (T.d.S.F.F.)

4 Marine Biotechnology Program, Admiral Paulo Moreira Marine Research Institute (IEAPM), Brazilian Navy, Arraial do Cabo, Rio de Janeiro 28930-000, Brazil; thayssafagundes@id.uff.br (T.d.S.F.F.)

5 Food Engineering Department, Embrapa Agroindústria de Alimentos, Rio de Janeiro 23020-470, Brazil; virginia.matta@embrapa.br (V.M.d.M)

6 Post-Graduation Program in Translational Drugs and Medicines, Institute of Technology in Medicines, Oswaldo Cruz Foundation, Rio de Janeiro 21040-900, Brazil

7 Laboratory of Organic and Inorganic Chemical Analysis, National Institute of Technology, Rio de Janeiro 20081-312, Brazil; claudete.kunigami@int.gov.br (C.N.K.); eliane.jung@int.gov.br (E.P.J.)

* Correspondence: davysonmoreira@jbrj.gov.br (D.d.L.M.); leilson.oliveira@int.gov.br (L.d.O.R.)

Table S1: Pearson' Correlation for results of experimental design.

Variable	Correlations (Spreadsheet1) Marked correlations are significant at p <0.050 n=17			
	TPC	$ABTS^{\bullet+}$	$DPPH^{\bullet}$	FRAP
TPC	1.0000 p= ---	0.9761 p=0.000	0.9821 p=0.000	0.9733 p=0.000
$ABTS^{\bullet+}$	0.9761 p=0.000	1.0000 p= ---	0.9825 p=0.000	0.9910 p=0.000
$DPPH^{\bullet}$	0.9821 p=0.000	0.9825 p=0.000	1.0000 p= ---	0.9914 p=0.000
FRAP	0.9733 p=0.000	0.9910 p=0.000	0.9914 p=0.000	1.0000 p= ---

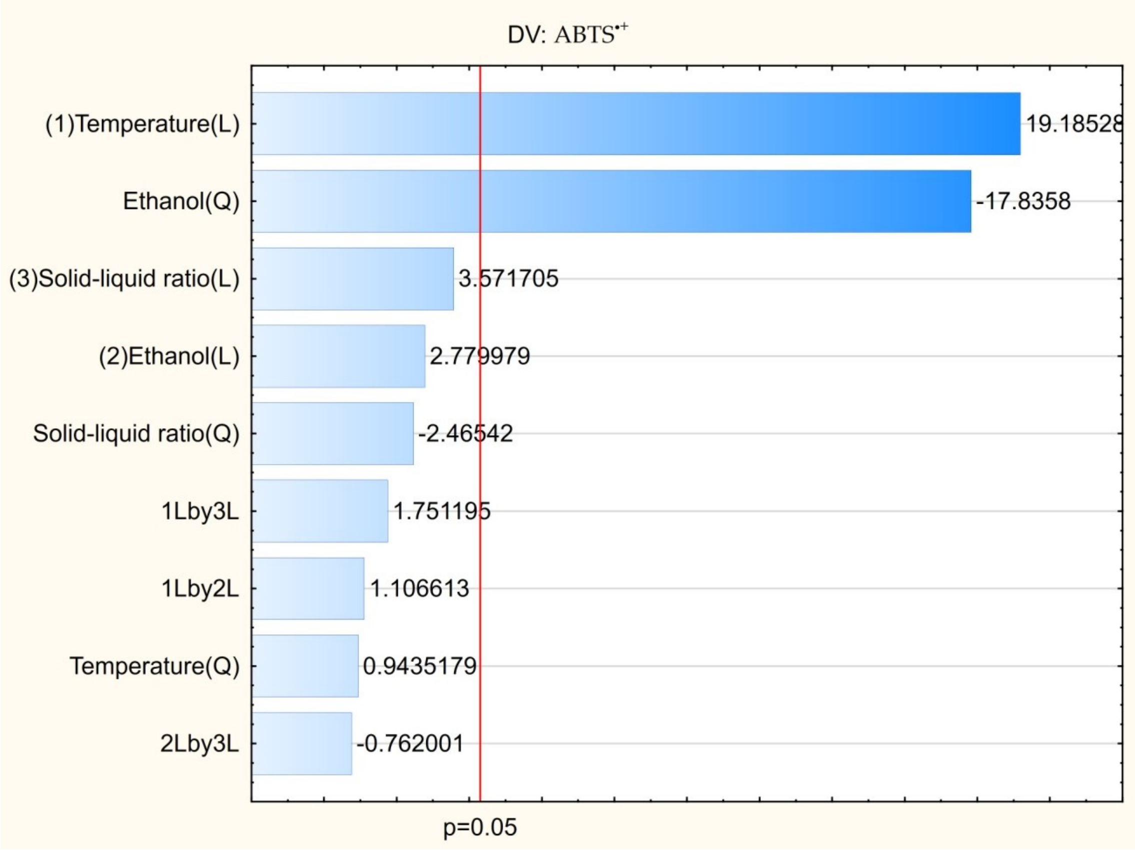

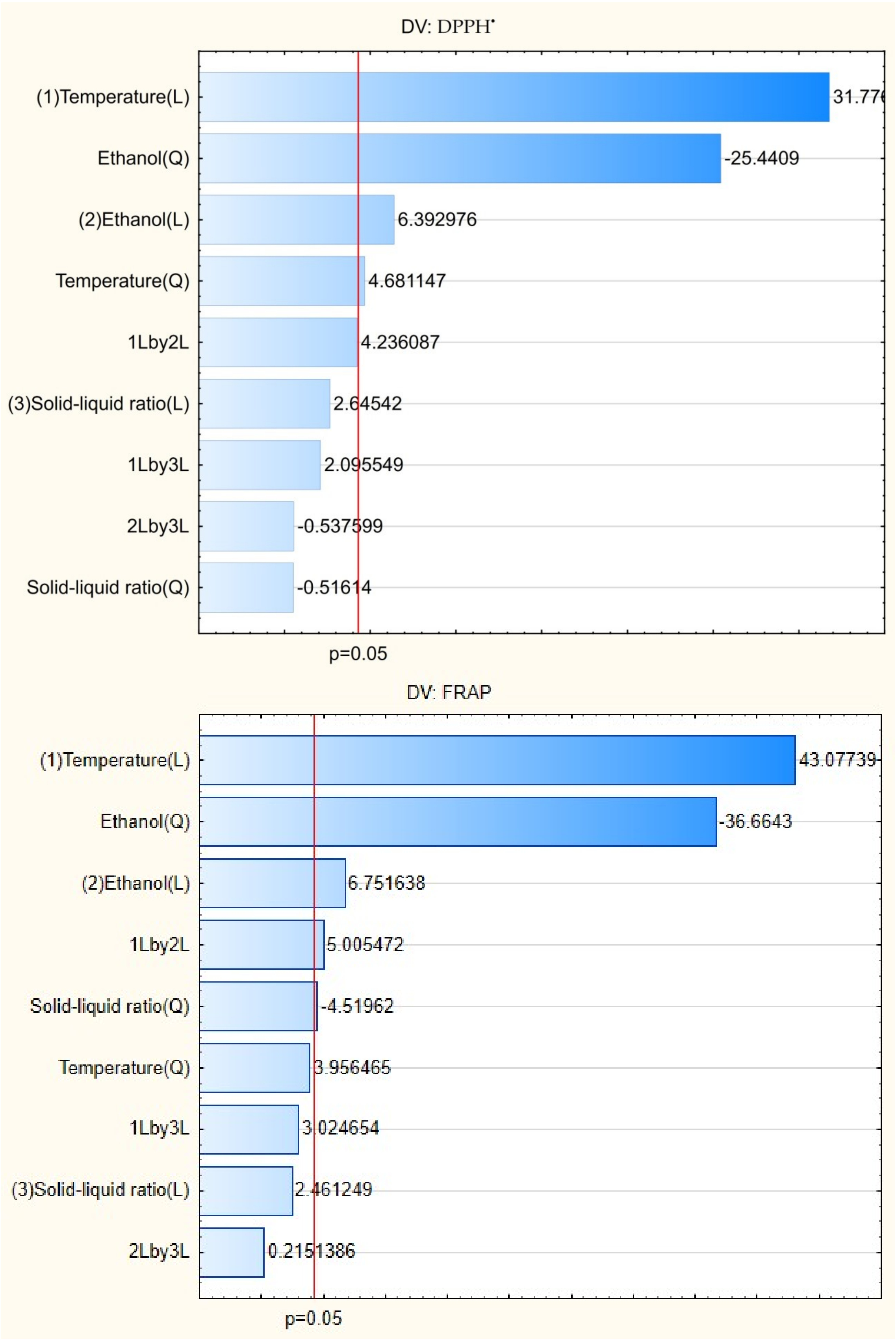

Figure S1 - Influence of the independent variables on the antioxidant capacity of the extract of *Passiflora cincinnata* defatted seeds.

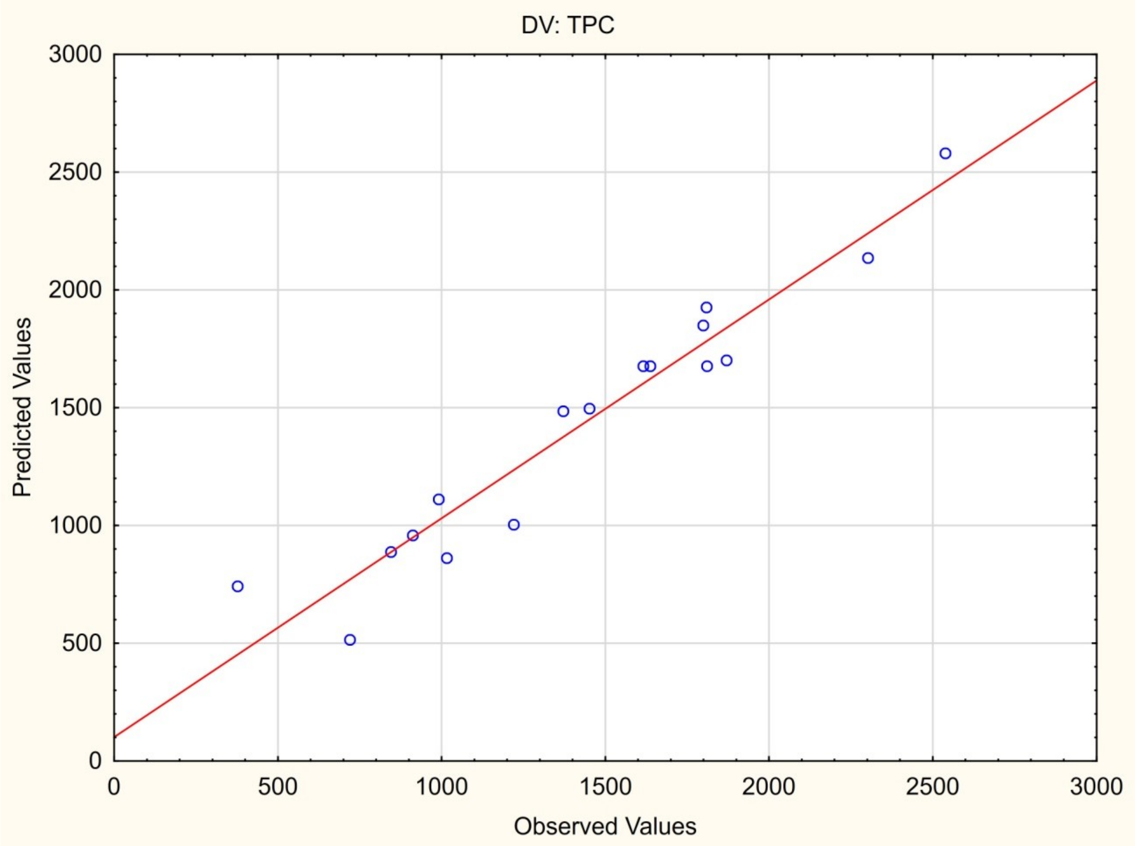
DV: TPC
Predicted Values
Observed Values
0
500
1000
1500
2000
2500
3000

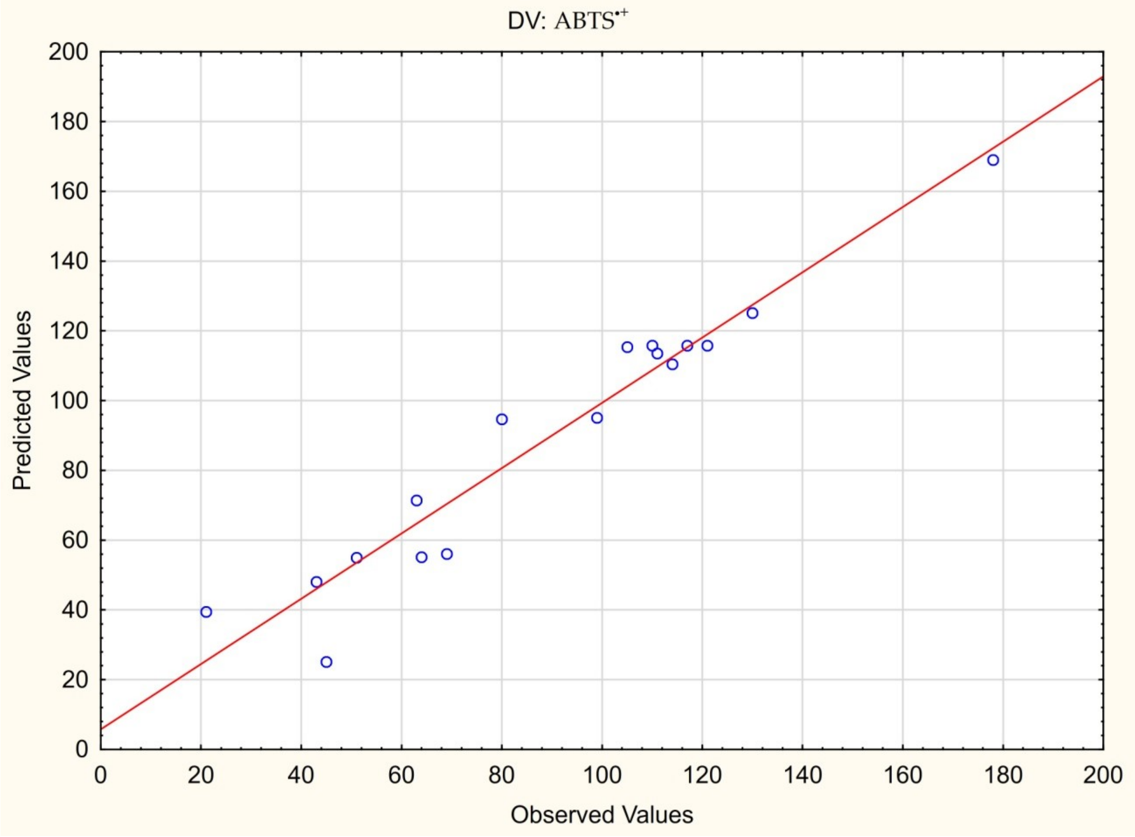
DV: ABTS•+
Predicted Values
Observed Values
0
20
40
60
80
100
120
140
160
180
200

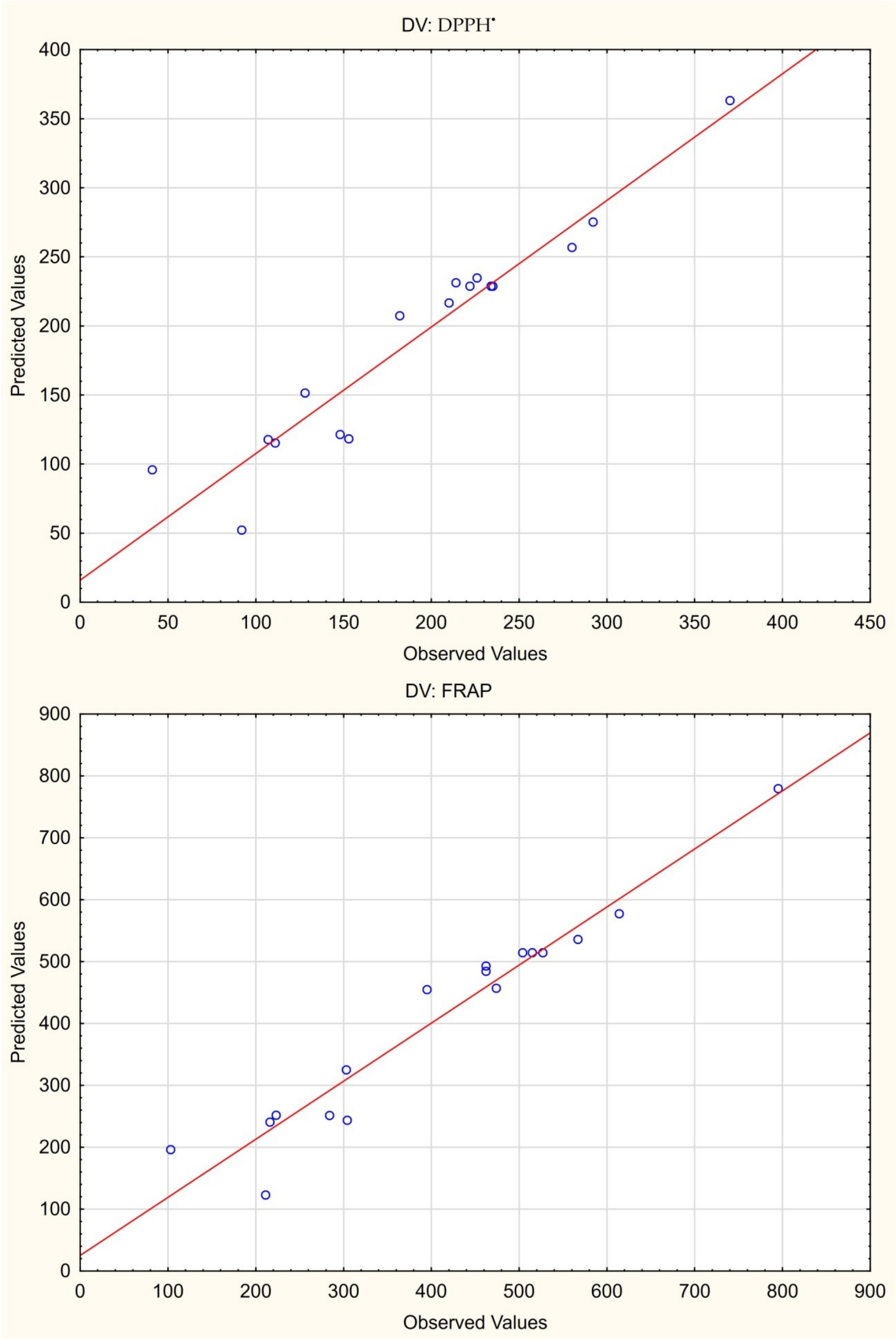

Figure S2 – Precicted versus observed values for responses of the experimental design (TPC – Total phenolic compounds and antioxidant capacity by $ABTS^{\bullet +}$, $DPPH^{\bullet}$ and FRAP assays).
